# Supplementary material for: MetaRibo-Seq measures translation in microbiomes
Source: Nat Commun. 2020 Jun 29;11:3268. doi: 10.1038/s41467-020-17081-z (PMC7324362; doi:10.1038/s41467-020-17081-z)
Supplement: Supplementary file 10 — Supplementary Data 7 [file 41467_2020_17081_MOESM10_ESM.zip › File2/Confidence_VeryHigh_Taxonomy/34611_out.krona.html]

Javascript must be enabled to view this page.

members
magnitude
magnitudeUnassigned
count
unassigned
taxon
rank

34611\_out

4

superkingdom
2
4

976
4
phylum

class
200643
4

4
171549
order

family
4
171552

genus
838
4

1947107
1
species

SRS019808\_contig\_number\_contig-100\_10793.91253

3
1262930

SRS015431\_contig\_number\_76908SRS143070\_contig\_number\_11091SRS147271\_contig\_number\_14887
species
